# miR-18a increases insulin sensitivity by inhibiting *PTEN*

**DOI:** 10.18632/aging.202319

**Published:** 2020-12-03

**Authors:** Yongqiang Zhou, Ruoqi Wu, Huafang Su, Kejie Li, Chun Chen, Raoying Xie

**Affiliations:** 1 Department of Radiation and Medical Oncology, The First Affiliated Hospital of Wenzhou Medical University, Wenzhou 325000, Zhejiang, China; 2 Department of Orthopedics, The First Affiliated Hospital, Wenzhou Medical University, Wenzhou 325000, Zhejiang, China

**Keywords:** miR-18a, insulin sensitivity, PTEN, T2DM

## Abstract

The miR-17-92 cluster (miR-17, miR-18a, miR-19a, miR-20a, miR-19b-1 and miR-92a) contributes to the occurrence and development of various diseases by inhibiting multiple target genes. Here, we explored the effects of miR-18a on insulin sensitivity. Quantitative real-time PCR indicated that serum miR-18a levels were lower in type 2 diabetes mellitus patients than in healthy controls, suggesting that miR-18a may influence blood glucose levels. Global overexpression of miR-18a in transgenic mice increased their glucose tolerance and insulin sensitivity, while it reduced expression of the phosphatase and tensin homolog deleted on chromosome ten (PTEN) in their skeletal muscle and adipose tissue. Western blotting indicated that overexpressing miR-18a in 3T3-L1 and C2C12 cells enhanced insulin-stimulated AKT phosphorylation and suppressed PTEN expression, while inhibiting miR-18a had the opposite effects. These results suggest that miR-18a improves insulin sensitivity by downregulating *PTEN*. This makes miR-18a a potentially useful target for the treatment of diabetes mellitus in the future.

## INTRODUCTION

Across the world, type 2 diabetes mellitus (T2DM) is a prevalent chronic disease [1] that seriously threatens human health [2]. The critical contributors to the pathogenesis of T2DM include insulin resistance (which impairs glucose disposal in skeletal muscle, adipocytes and the liver), inactivity, increasing obesity rates and advancing age [3]. However, the pathogenesis of T2DM is not fully understood.

microRNAs (miRNAs) influence the sensitivity of tissues to insulin, the activity of pancreatic β-cells, the development of the pancreas and the pathogenesis of T2DM [4–6]. For instance, miR-103/miR-107, miR-802 and miR-143 have been demonstrated to reduce insulin sensitivity in mice [7–9]. In addition, miR-375 and miR-9 have been shown to reduces insulin secretion, and miR-34a and miR-375 are involved in the development of pancreas [10–12]. However, further research is needed to determine how miRNAs alter blood glucose levels, and to identify miRNAs that promote insulin sensitivity and glucose metabolism, in order to discover potential therapeutic targets for T2DM.

The polycistronic miR-17-92 cluster includes six miRNAs—miR-17, miR-18a, miR-19a, miR-19b-1, miR-20a and miR-92a—and regulates multiple cellular processes that are involved in proliferation, oncogenesis, differentiation, angiogenesis, survival and blood glucose control [13, 14]. The miRNAs in this cluster can work independently or cooperatively to influence the occurrence, development and prognosis of various diseases; for example, miR-19 has been confirmed to drive the tumorigenicity of the miR-17-92 cluster in MYC-induced B-cell lymphoma [15]. However, the involvement of miR-18a in blood glucose regulation is not clear.

Phosphatase and tensin homolog deleted on chromosome ten (PTEN), a well-known tumor suppressor and major inhibitor of the phosphoinositide 3-kinase (PI3K)/AKT pathway, has also been reported to be an important regulator of glucose and lipid metabolism. Although PTEN deficiency induces multiple forms of cancer, it can also trigger a series of metabolic alterations that effectively enhance insulin sensitivity. Moreover, several studies have demonstrated that *PTEN* polymorphisms are associated with insulin resistance [16–18].

In the present study, we assessed serum miR-18a levels in T2DM patients, and examined the effects of miR-18a on PTEN expression and insulin sensitivity.

## RESULTS

### Downregulation of miR-18a in serum from T2DM patients

**Figure 1:**
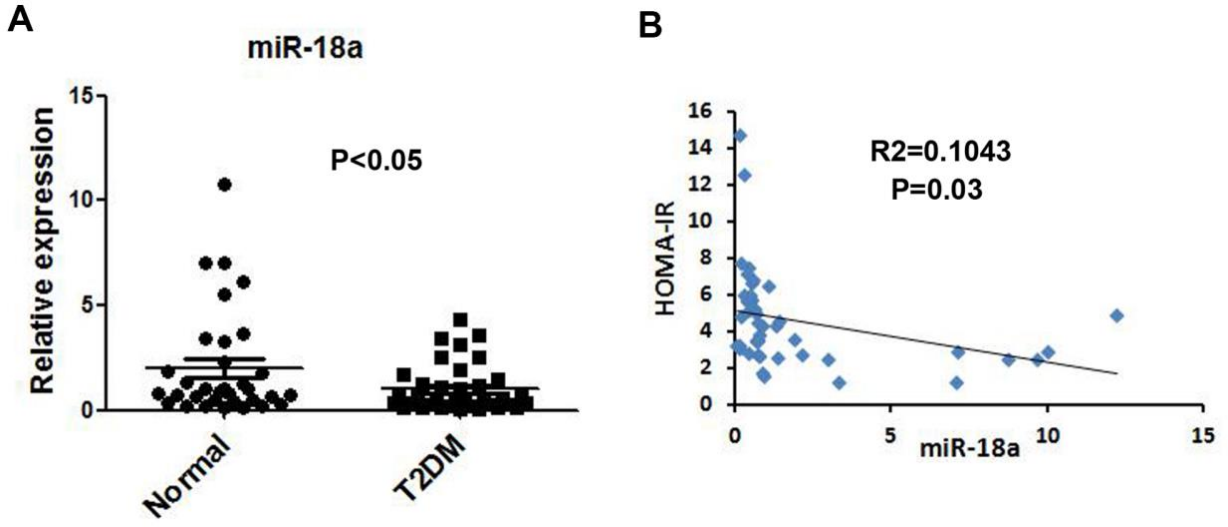
**Downregulation of miR-18a in serum from T2DM patients.** (**A**) Basal miR-18a levels in healthy subjects (n=44) and T2DM patients (n=49) were detected using qRT-PCR. (**B**) Correlation between miR-18a levels and homeostasis model assessment of insulin resistance (HOMA-IR) values. P values <0.05 were considered statistically significant.

miR-146a was previously reported to be downregulated in serum [19] and peripheral blood mononuclear cells [20] from T2DM patients (Supplementary Figure 1); thus, we used miR-146a as a reference to assess serum miR-18a levels. Quantitative real-time PCR (qRT-PCR) indicated that serum miR-18a levels were lower in T2DM patients than in normal controls (Figure 1A). Moreover, miR-18a levels correlated negatively with homeostasis model assessment of insulin resistance values (R^2^=0.1043, P=0.03, Figure 1B). These findings strongly suggested that miR-18a influences insulin function and glucose homeostasis.

### Upregulation of miR-18a enhances glucose metabolism and insulin sensitivity in RL-18a mice

**Figure 2:**
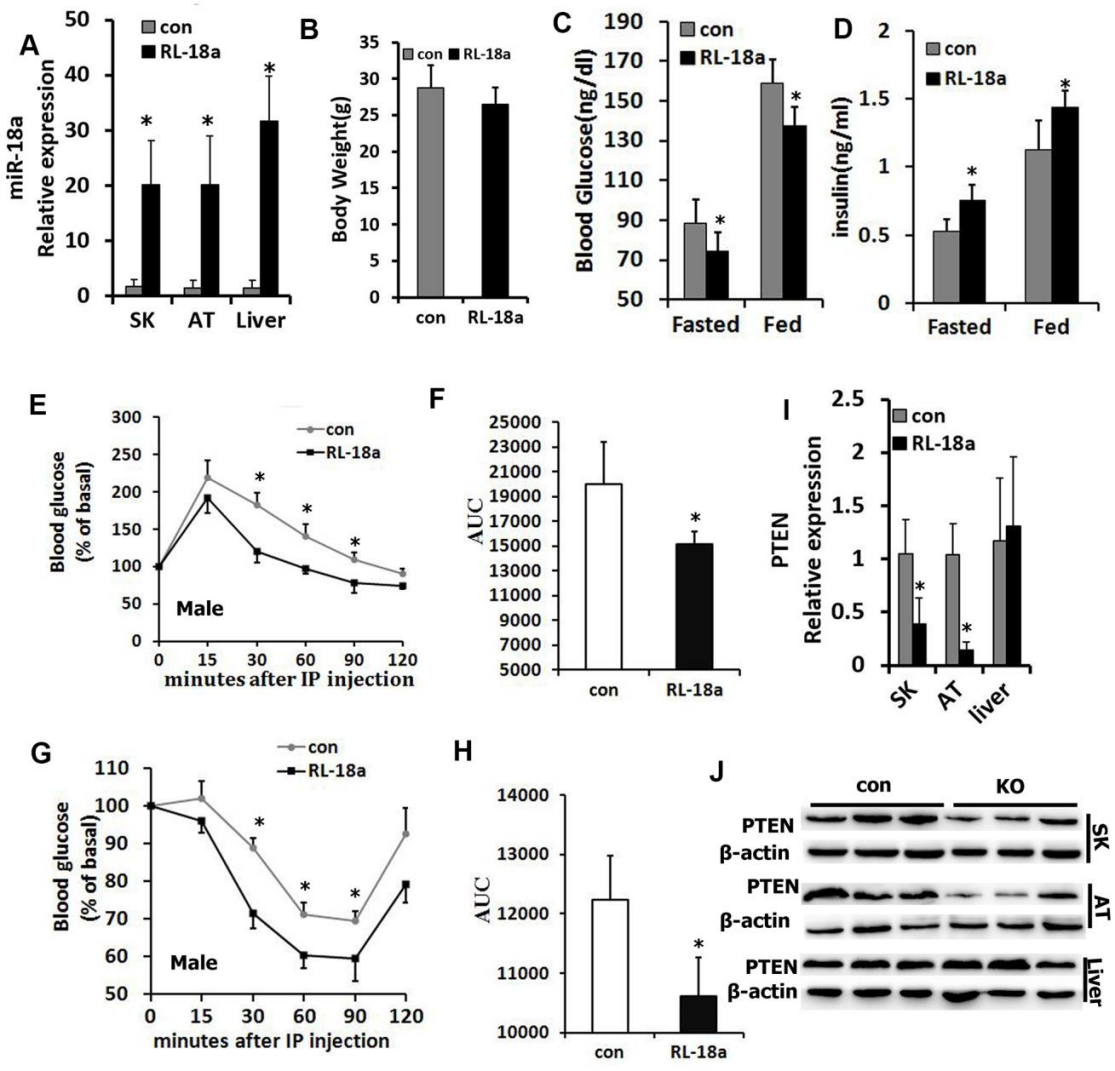
**Overexpression of miR-18a enhances glucose metabolism and insulin sensitivity in male RL-18a mice.** (**A**) MiR-18a transgene levels in multiple tissues from RL-18a mice were determined using qRT-PCR. (**B**) Body weights of RL-18a and control mice (n=7). (**C**) Blood glucose concentrations in fed and 12-hour-fasted mice at different times. (**D**) Serum insulin concentrations in fed and 12-hour-fasted mice. (**E**, **F**) Glucose tolerance test results determined with an enzyme-linked immunosorbent assay in 12-hour-fasted mice (**E**), and the area under the curve (AUC) for this test (**F**). (**G**, **H**) Insulin tolerance test in 12-hour-fasted mice (**G**), and the AUC for this test (**H**). (**I**) *PTEN* expression in skeletal muscle (SK), adipose tissue (AT) and liver samples from RL-18a mice, assessed using qRT-PCR. (**J**) PTEN expression in SK, AT and liver samples from RL-18a mice, assessed using Western blotting. n=7 male mice/group.

Next, we used a Cre/loxP system to overexpress miR-18a in mice (‘RL-18a mice’). Using qRT-PCR, we confirmed that miR-18a was overexpressed in skeletal muscle, adipose tissue and liver samples from male RL-18a mice (Figure 2A). miR-18a overexpression did not alter the final body weights of male RL-18a mice (Figure 2B).

In both the fasting and fed states in males, blood glucose levels were lower in RL-18a mice than in control mice, while circulating insulin levels were higher in RL-18a mice than in control mice (Figure 2C, 2D). Glucose tolerance tests in both males and females indicated that the glucose clearance efficiency was higher in RL-18a mice than in control mice (Figure 2E, 2F; Supplementary Figure 2A, 2B), suggesting that RL-18a mice have an enhanced glucose tolerance. To evaluate the insulin sensitivity of peripheral tissues, we also conducted insulin tolerance tests. In both males and females, RL-18a mice exhibited greater insulin sensitivity than control mice (Figure 2G, 2H; Supplementary Figure 2C, 2D).

PTEN is a known negative regulator of insulin sensitivity [21]; thus, we used qRT-PCR (Figure 2I) and Western blotting (Figure 2J) to analyze PTEN expression in skeletal muscle, adipose tissue and liver samples from male RL-18a mice. As expected, PTEN levels in skeletal muscle and adipose tissue were lower in RL-18a mice than in control mice; however, there were no obvious changes in PTEN levels in the liver.

### miR-18a enhances insulin-induced AKT phosphorylation/activation in insulin target cells

**Figure 3:**
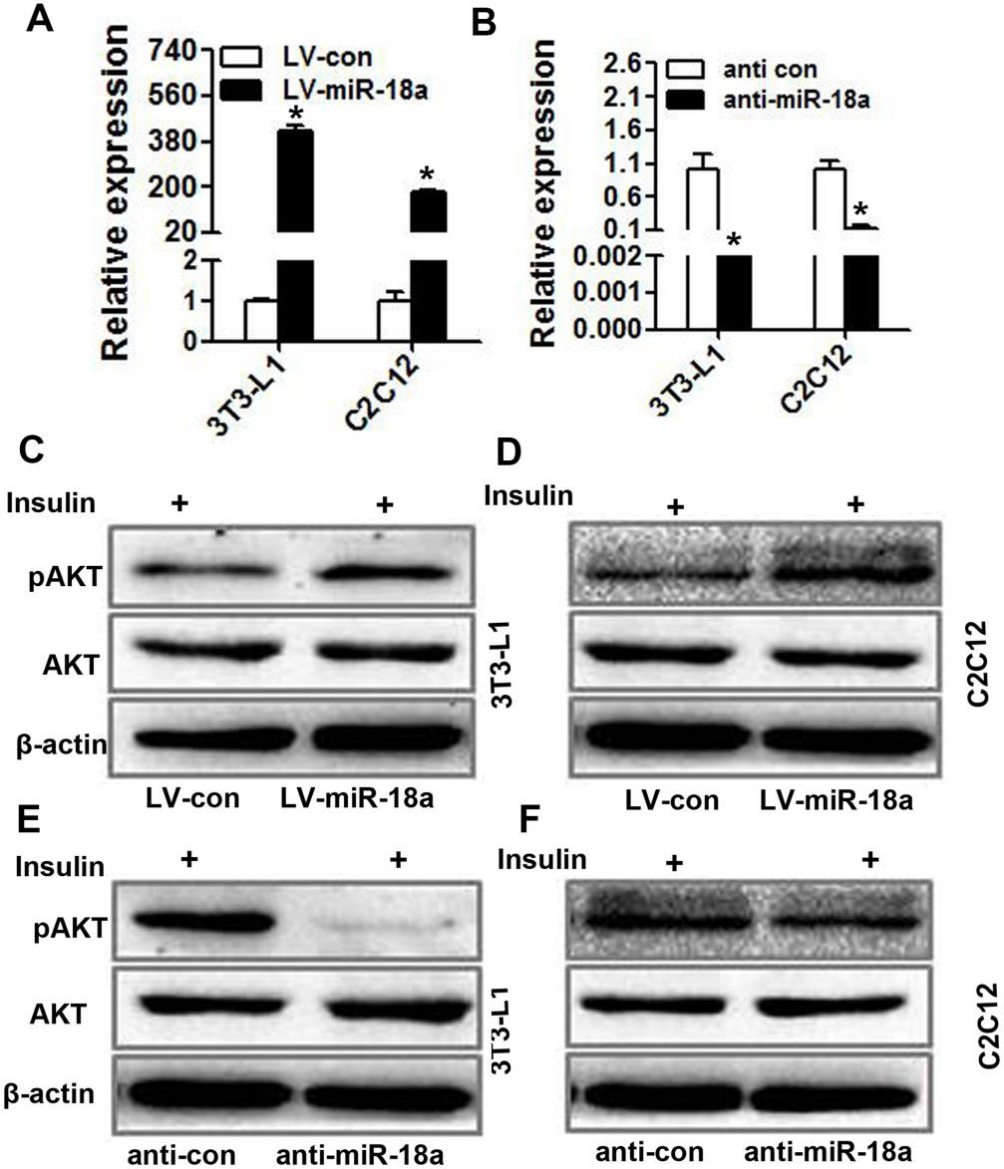
**miR-18a enhances insulin-stimulated AKT phosphorylation/activation in insulin target cells.** (**A**, **B**) MiR-18a levels were detected using qRT-PCR in 3T3-L1 and C2C12 cells in which miR-18a was overexpressed (LV-miR-18a) (**A**) or inhibited (anti-miR-18a) (**B**). (**C**, **D**) Western blot analysis of insulin-stimulated AKT phosphorylation in 3T3-L1 (**C**) and C2C12 (**D**) cells overexpressing miR-18a. (**E**, **F**) Western blot analysis of insulin-stimulated AKT phosphorylation in 3T3-L1 (**E**) and C2C12 (**F**) cells transfected with miR-18a inhibitors (mean ± SD, *P<0.05).

Insulin activity and sensitivity depend on PI3K-AKT pathway activation [22]. To further investigate the function of miR-18a in insulin target cells, we transiently transfected 3T3-L1 preadipocytes and C2C12 myoblasts with miR-18a mimics or inhibitors (‘anti-miR-18a’). The upregulation and inhibition of miR-18a expression in these cells was confirmed using qRT-PCR (Figure 3A, 3B). Western blotting indicated that the insulin-induced phosphorylation of AKT increased in 3T3-L1 and C2C12 cells transfected with miR-18a mimics (Figure 3C, 3D) but decreased in 3T3-L1 and C2C12 cells transfected with miR-18a inhibitors (Figure 3E, 3F). These results verified that miR-18a can enhance insulin sensitivity.

### miR-18a binds directly to the 3’ untranslated region (UTR) of *PTEN*

**Figure 4:**
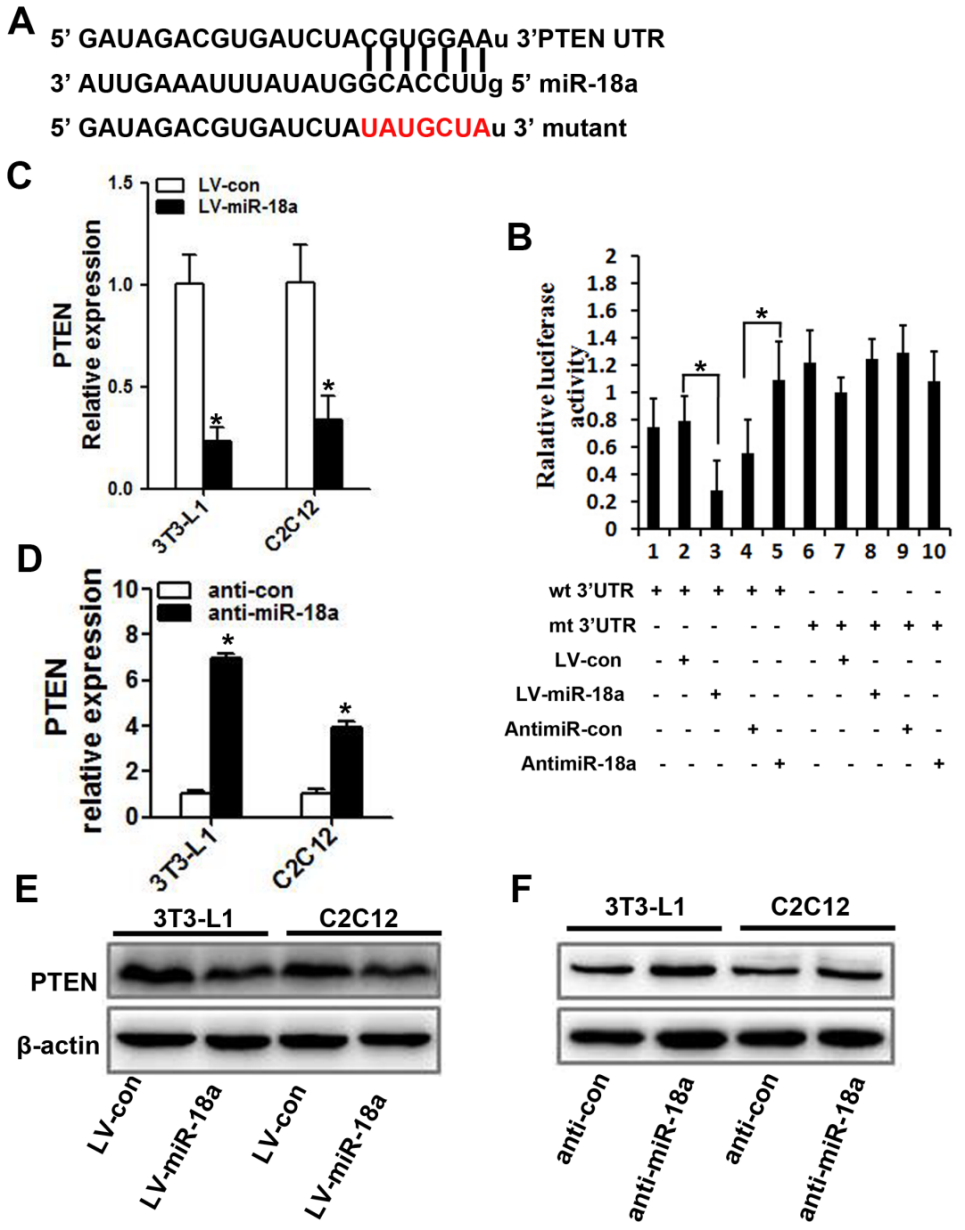
***PTEN* is a target gene of miR-18a.** (**A**) The predicted binding sequence for miR-18a within the human *PTEN* 3’UTR. Seed sequences are highlighted. (**B**) Luciferase reporter assay in 3T3-L1 cells. The bar graph displays the mean ± SD of three independent transfection experiments. *P<0.05. (**C**, **D**) *PTEN* mRNA levels in 3T3-L1 and C2C12 cells transfected with LV-miR-18a, anti-miR-18a or the corresponding controls (LV-con and anti-con, respectively). (**E**, **F**) PTEN protein levels were assessed using Western blotting in 3T3-L1 and C2C12 cells transfected with LV-miR-18a, LV-con, anti-miR-18a or anti-con.

Next, we searched for potential targets of miR-18a, and found that its sequence complemented the 3’UTR of *PTEN* (Figure 4A). Accordingly, the mRNA and protein levels of PTEN decreased when miR-18a was overexpressed (Figure 4C, 4E) and increased when miR-18a was inhibited (Figure 4D, 4F) in 3T3-L1 and C2C12 cells.

To determine whether miR-18a enhanced the insulin sensitivity of insulin target cells by directly inhibiting *PTEN*, we subcloned wild-type or mutant miR-18a binding sequences from the *PTEN* 3’UTR (‘wt 3’UTR’ and ‘mt 3’UTR,’ respectively) into luciferase reporter vectors (Figure 4A). Then, we co-transfected 3T3-L1 cells with one of these vectors and either miR-18a mimics or inhibitors. In 3T3-L1 cells transfected with the *PTEN* wt 3’UTR, the luciferase activity was significantly lower in those co-transfected with miR-18a mimics than in those co-transfected with the control vector (Figure 4B, lanes 2 and 3; P<0.05). On the other hand, co-transfection with anti-miR-18a increased the luciferase activity of the *PTEN* wt 3’UTR vector approximately 1.5-fold compared with the corresponding control (Figure 4B, lanes 4 and 5; P<0.05). However, no such changes were observed in cells co-transfected with the *PTEN* mt 3’UTR vector and either miR-18a mimics or inhibitors (Figure 4B, lanes 7 and 8, lanes 9 and 10). These results confirmed that miR-18a binds directly to *PTEN* in insulin target cells.

### Overexpression of *PTEN* reverses the miR-18a-induced phosphorylation of AKT

**Figure 5:**
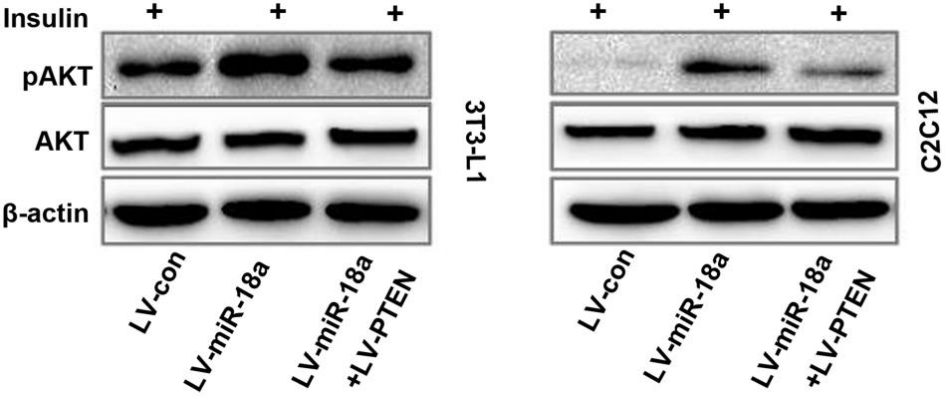
**Functional recovery test confirming that *PTEN* overexpression reverses miR-18a-induced AKT phosphorylation.** Western blot analysis of insulin-stimulated AKT phosphorylation in 3T3-L1 and C2C12 cells transfected with different plasmids.

Finally, we evaluated whether ectopic *PTEN* expression would reverse the miR-18a-induced phosphorylation of AKT in insulin-treated cells. As anticipated, *PTEN* overexpression suppressed AKT phosphorylation in 3T3-L1 and C2C12 cells treated with miR-18a mimics (Figure 5). These results suggested that miR-18a enhances insulin sensitivity in insulin target cells by inhibiting *PTEN*.

## DISCUSSION

Receptor tyrosine kinases (including the insulin receptor) are well known to activate the PI3K/AKT signaling pathway, which then can induce cell proliferation, differentiation, migration and survival [22]. Tight regulation and tissue specificity determine how cells respond to PI3K/AKT pathway activation [23, 24]. The PI3K/AKT signaling pathway facilitates various activities of insulin, such as promoting skeletal muscle and adipose tissue glucose uptake and glycogenesis and reducing hepatic glycogen breakdown and glucose release [25, 26].

Previous studies have shown that the miR-17-92 cluster of genes is involved in embryo differentiation, is widely expressed in lymph, breast, liver, testis, intestinal, pancreatic and other tissues, and is upregulated in some tumor tissues [27, 28]. In addition, recent studies have indicated that miR-17-92 may regulate blood glucose levels [29, 30]. The miR-17-92 gene cluster includes six components—miR-17, miR-18a, miR-19a, miR-20a, miR-19b-1 and miR-92a—some of which are involved in the pathogenesis of diabetes. For example, miR-19b-1 can inhibit *NeuroD1* expression, ultimately downregulating insulin 1 expression [31]. MiR-17, miR-20a and miR-92-1 levels were found to be reduced in diabetic heart disease [32]. While poor adipocyte differentiation promotes insulin resistance, the miR-17-92 gene cluster can significantly accelerate the differentiation of adipocyte precursor cells [33]. These results indicate that the miR-17-92 gene cluster may be associated with diabetes and islet cell function; however, the clinical significance and function of each component need to be further studied. Since little research has examined the effects of miR-18a on glucose metabolism, we selected this miRNA as the focus of our study.

The target tissues of insulin are skeletal muscle, adipose tissue and liver; thus, we measured the expression of PTEN in these tissues. However, in our analyses of RL-18a mice, we did not find that *PTEN* was a target of miR-18a in the liver. To elucidate the mechanism responsible for insulin sensitization in insulin target cells, we performed *in vitro* studies in 3T3-L1, C2C12 and HepG2 cells. These results suggested that miR-18a does not improve insulin sensitivity by inhibiting *PTEN* in the liver. Previous research has shown that one miRNA may have several target genes, while several miRNAs can inhibit a particular mRNA [34], contributing to the complexity of post-transcriptional regulation [35, 36]. Therefore, we speculate that other miRNAs inhibit *PTEN* in the liver. Moreover, in view of the tissue specificity of certain miRNAs [37], we postulate that miR-18a does not function in the liver. In addition, the miR-17-92 cluster and the paralogous miR-106b-25 cluster have been shown to cooperate during embryonic development [38] and to inhibit some of the same mRNAs [14]. Therefore, the component miRNAs of these two gene clusters may also cooperate in the liver. However, further experiments are needed to determine why miR-18a did not alter insulin sensitivity in the liver by inhibiting *PTEN*.

In conclusion, miR-18a enhanced insulin sensitivity by inhibiting *PTEN* in 3T3-L1 and C2C12 cells. Given that the downregulation of PTEN is known to increase insulin sensitivity [18, 39], miR-18a overexpression may be a beneficial strategy to restore insulin function in patients with T2DM.

## MATERIALS AND METHODS

### Establishment of miR-18a transgenic mice

Wild-type FVB/N mice and homozygous EIIa-Cre transgenic mice (FVB/N-Tg (EIIa-Cre) C5379Lmgd/J) were purchased from the Model Animal Research Center of Nanjing University. miR-18a was overexpressed in RL-18a transgenic mice using a Cre/loxP system on a C57BL/6 background, as reported previously [40]. All procedures involving animals adhered to the Guide for the Care and Use of Laboratory Animals of Wenzhou Medical University. The mice were anesthetized with sodium pentobarbital during all surgeries, and the suffering of the animals was minimized.

### Patients and healthy subjects

Samples were collected from the Department of Endocrinology of the Second Affiliated Hospital of Guangzhou Medical University. The Hospital Ethics Committee of the Second Affiliated Hospital of Guangzhou Medical University approved this study. Serum samples from healthy subjects (n=44) and patients with a new diagnosis of T2DM (n=49) were analyzed using qRT-PCR. T2DM was diagnosed based on the diagnostic standards of the World Health Organization [41]. The healthy subjects had no familial diabetes history, and their fasting plasma glucose levels were <6.1 mM, while their two-hour post-load plasma glucose concentrations after a 75-g oral glucose tolerance test were <7.8 mM. Clinical data for the patients and controls are shown in Supplementary Table 1.

### Metabolic studies

For the glucose tolerance test, male and female mice received an intraperitoneal injection of glucose (2 g/kg body weight) after a 12-hour overnight fast. For the insulin tolerance test, male and female mice received an intraperitoneal injection of human insulin (Novo Nordisk; 0.75 IU/kg body weight) after a 6-hour overnight fast. The blood glucose levels in the tail veins of the mice prior to and at various time points after the respective injections were analyzed with an automatic blood glucose meter (One Touch Lifescan, Johnson & Johnson, USA). The insulin concentrations in serum samples were detected with an enzyme-linked immunosorbent assay in accordance with the manufacturer’s recommendations (Millipore Rat/Mouse Insulin ELISA Kit, EZRMI-13K), as previously described [40].

### qRT-PCR

The miRNAs in serum samples from T2DM patients and healthy controls were extracted using a microRNA RNeasy Mini Kit (Exiqon) according to the vendor’s instructions; the product information is listed in Supplementary Table 2. Trizol, an iScript cDNA synthesis kit and a SYBR PrimeScript miRNA RT-PCR Kit were obtained from TaKaRa Bio. RNA was isolated, reverse transcribed into cDNA and assessed via qRT-PCR in accordance with the manufacturer’s protocols and our prior publication [42]. The primers used to amplify *PTEN* (Invitrogen, Shanghai, China) were: 5’-TGGCATTTGCTGAACGCATTT-3’ (forward) and 5’- TGCAGCCAGGTCTAATTGTTTT-3’ (reverse). Other primers are reported in Supplementary Table 2. The results were calculated using the relative quantification protocol (2^-ΔΔCt^).

### Western blot analysis

The Western blotting procedures were detailed in our previous report [42]. The antibodies used in this study are shown in Supplementary Table 3.

### Luciferase assay

A dual-luciferase reporter assay kit was obtained from Promega Corporation (USA). The experiments were performed according to the manufacturer’s directions and a previous report [43].

### Statistical analysis

Data were analyzed with SPSS 18.0 software. All data are shown as the mean ± standard deviation (SD). Values were deemed statistically significant at *P<0.05.

## Supplementary Material

Supplementary Figures

Supplementary Tables
