# Supplementary material for: miR-18a increases insulin sensitivity by inhibiting PTEN
Source: Aging (Albany NY). 2020 Dec 3;13(1):1357–68. doi: 10.18632/aging.202319 (PMC7835052; doi:10.18632/aging.202319)
Supplement: Supplementary Figures [file aging-13-202319-s001.pdf]

SUPPLEMENTARY FIGURES

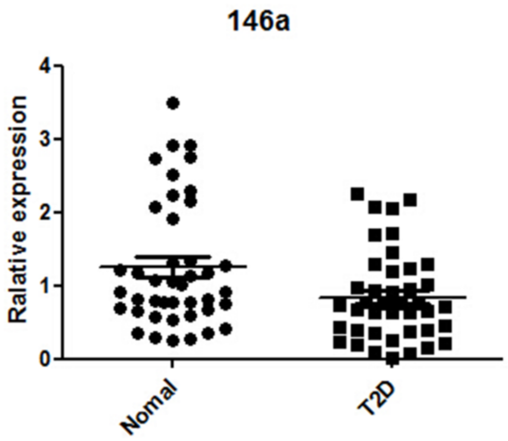

Supplementary Figure 1. The expression of miR-146a in T2DM.

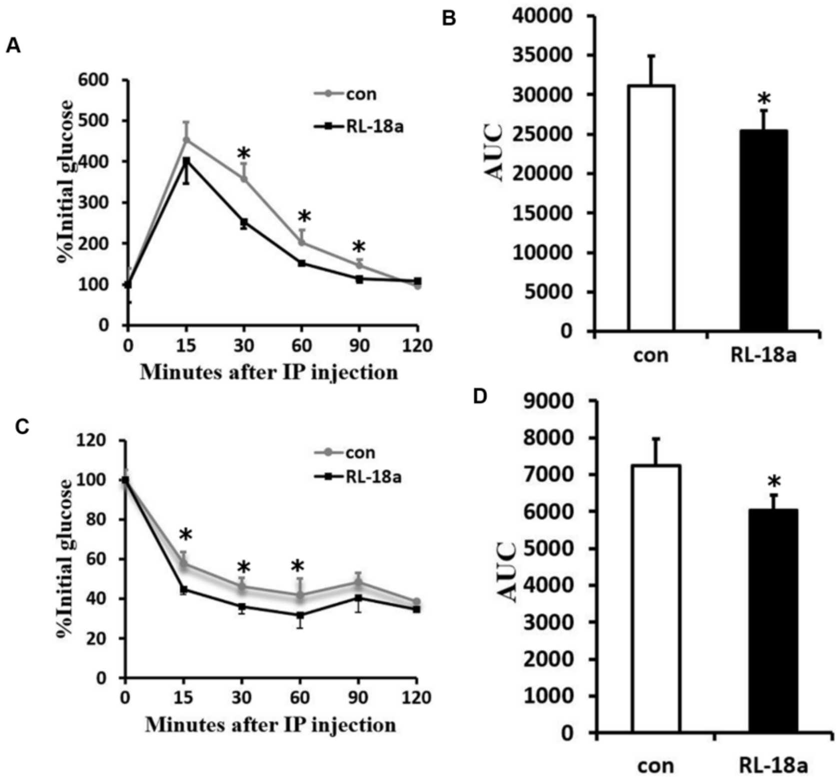

Supplementary Figure 2. RL-18a female mice displayed improved glucose tolerance and enhanced insulin sensitivity. (**A**, **B**) GTT in 12-hour–fasted female mice (**A**) and AUC for this GTT (**B**). (**C**, **D**) ITT in 12-hour–fasted female mice (**C**) and AUC for this ITT (**D**). RL-18a female mice (*n = 6*) vs control female *mice* (*n* = 6).
