# Supplementary material for: miR-18a increases insulin sensitivity by inhibiting PTEN
Source: Aging (Albany NY). 2020 Dec 3;13(1):1357–68. doi: 10.18632/aging.202319 (PMC7835052; doi:10.18632/aging.202319)
Supplement: Supplementary Tables [file aging-13-202319-s002.pdf]

SUPPLEMENTARY TABLES

Supplementary Table 1. Baseline clinical features of the patients.

Characteristics	Nomal mean±SD	T2DM mean±SD	P-value
Age(years)	50.8±17.1	55.4±12.0	0.136
Fasting plasma glucose (mmol/L)	5.3±0.6	9.8±3.5	0.000
2-hour postprandial plasma glucose (mmol/L)	6.6±1.2	16.8±5.5	0.000
Fasting insulin (mU/L)	9.8±5.2	10.6±4.7	0.420
2-hour postprandial insulin (mU/L)	72.3±68.0	40.4±27.8	0.005
Aspartate aminotransferase (U/L)	21.0±6.3	25.0±9.7	0.280
Alanine aminotransferase (U/L)	16.8±5.5	26.8±14.0	0.064
Creatinine (umol/L)	66.0±15.8	66.8±14.5	0.899
Triglyceride (mmol/L)	1.0±0.2	1.6±0.9	0.040
Cholesterol (mmol/L)	4.7±1.2	4.9±1.2	0.755
High density lipoprotein (mmol/L)	1.6±0.4	1.0±0.3	0.001
Low density lipoprotein (mmol/L)	3.0±0.95	4.3±1.5	0.113
Uric acid (umol/L)	350.1±62.1	352.8±94.5	0.941

Supplementary Table 2. Product information.

Purpose	Primers and kits	No.(EXIQON)
The detection of target	has-miR-18a-5p	204207
microRNAs	has-miR-146a-5p	204688
cDNA Synthesis Kit	ExiLENT SYBR® Green master mix 20ml	203420
qRT-PCR Kit	miRCURY RNA Isolation Kit – Biofluids (50)	300112
Isolation Kit	RNA-Spike-in, UniRT	203203

Supplementary Table 3. Primer sequences for each gene examined by qRT-PCR.

Primer name	Primer sequence
mmiR-18a RT primer	GTCGTATCCAGTGCAGGGTCCGAGGTATTCGCACTGGATACGACctatc
mmiR-18a forward primer	TAAGGTGCATCTAGTGCAG
universal reverse primer	GTGCAGGGTCCGAGGTATTC
RNU6-1 RT primer	AACGCTTCACGAATTTGCGT
RNU6-1 forward primer	CTCGCTTCGGCAGCACA
RNU6-1 reverse primer	AACGCTTCACGAATTTGCGT
